# Crystal structure and absolute configuration of (3a*R*,3′a*R*,7a*S*,7′a*S*)-2,2,2′,2′-tetra­methyl-3a,6,7,7a,3′a,6′,7′,7′a-octa­hydro-4,4′-bi[1,3-benzodioxol­yl], obtained from a Pd-catalyzed homocoupling reaction

**DOI:** 10.1107/S2056989016019927

**Published:** 2017-01-01

**Authors:** Mario A. Macías, Enrique Pandolfi, Valeria Schapiro, Gustavo P. Silveira, Guilherme D. Vilela, Leopoldo Suescun

**Affiliations:** a Departamento de Química, Universidad de los Andes, Carrera 1 No 18A-12, Bogotá, Colombia; b Departamento de Química Orgánica, Facultad de Química, Universidad de la República, Montevideo, Uruguay; c Departamento de Química Orgánica, Instituto de Química, Universidade Federal do Rio Grande do Sul, Porto Alegre/RS, 91501-970, Brazil; d Cryssmat-Lab/Cátedra de Física/DETEMA, Universidad de la República, Montevideo, Uruguay

**Keywords:** crystal structure, absolute configuration, homocoupling reaction, palladium-catalyzed, 1,3-benzodioxol­yl

## Abstract

The absolute configuration, *i.e.* (3a*R*,3′a*R*,7a*S*,7′a*S*), of the title compound, C_18_H_26_O_4_, synthesized *via* a palladium-catalyzed homocoupling reaction, was determined on the basis of the synthetic pathway and was confirmed by X-ray diffraction. The homocoupled mol­ecule is formed by two chemically identical moieties built up from two five- and six-membered fused rings. The supra­molecular assembly is controlled mainly by C—H⋯O inter­actions that lead to the formation of hydrogen-bonded chains of mol­ecules along the [001] direction, while weak dipolar inter­actions and van der Waals forces hold the chains together in the crystal structure.

## Abstract 2

The crystal structure of a homocoupled compound with absolute configuration 3a*R*,3′a*R*,7a*S*,7′a*S* was determined. The mol­ecule contains two similar moieties composed of two fused rings. Its supra­molecular structure is controlled mainly by C—H⋯O inter­actions.

## Chemical context   

**Figure 1:**
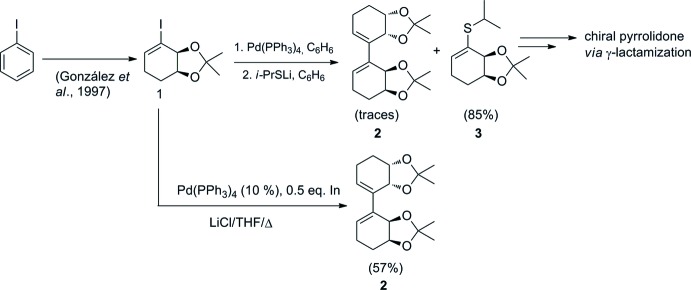
Synthetic pathway showing the formation of the homocoupled compound C_18_H_26_O_4_.

Over the last few years, we have focused our efforts on the synthesis of vinyl­sulfimines as precursors in γ-lactamization reactions to generate asymmetric pyrrolidone derivatives which are of inter­est in medicinal chemistry (Silveira *et al.*, 2012 ▸, 2014 ▸; Silveira & Marino, 2013 ▸; Pereira *et al.*, 2015 ▸). Encouraged by our previous experience in functionalizing halo-cyclo­hexa­diendiols (Heguaburu *et al.*, 2008 ▸; Labora *et al.*, 2010 ▸; Heguaburu *et al.*, 2010 ▸; Labora *et al.*, 2008 ▸), we synthesized a vinylic sulfide (mol­ecule **3** in Fig. 1 ▸) from protected iodo-cyclo­hexenediol (mol­ecule **1** in Fig. 1 ▸). This latter compound was obtained firstly by regioselective reduction of iodo­cyclo­hexa­dienediol derived from the biotransformation of iodo­benzene (González *et al.*, 1997 ▸). The obtained compound was treated with lithium iso­propyl­thiol­ate in the presence of 5% of Pd (PPh_3_)_4_ as catalyst to obtain the vinyl sulfide in 85% yield. Surprisingly, one of the attempts to perform this reaction proceeded to afford traces of the homocoupled product (mol­ecule **2** in Fig. 1 ▸). Considering this finding, we decided to prepare this new compound *via* a palladium-catalyzed homocoupling reaction of the vinylic iodide (mol­ecule **1** in Fig. 1 ▸), mediated by indium, according to the Lee protocol (Lee *et al.*, 2005 ▸). Herein, we report this new synthetic method and the crystal structure of the title compound.
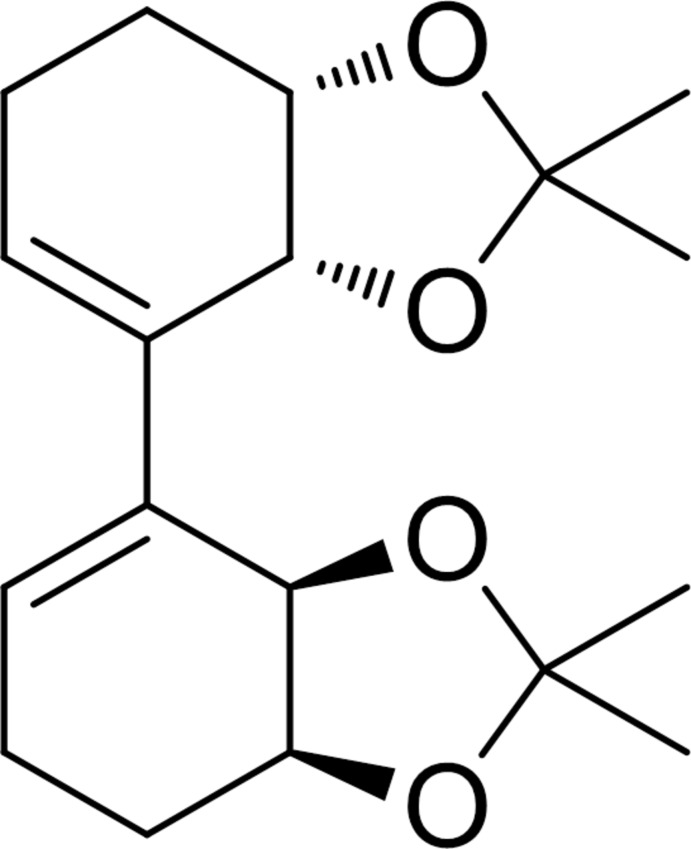


## Structural commentary   

**Figure 2:**
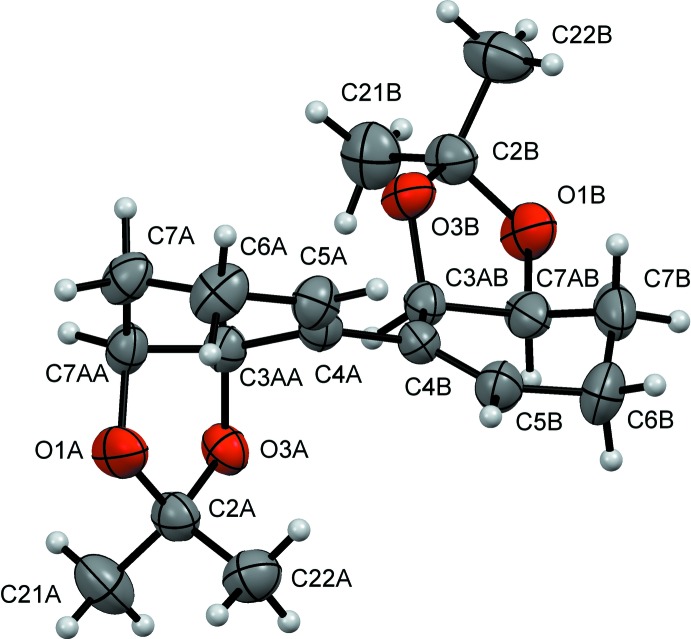
The mol­ecular structure of the title compound, showing anisotropic displacement ellipsoids drawn at the 50% probability level.

The absolute configuration of the title compound (Fig. 2 ▸) was determined to be 3a*R*,3′a*R*,7a*S*,7′a*S* by considering the synthetic pathway and confirmed by X-ray diffraction on the basis of the anomalous dispersion of light atoms only. The mol­ecule is built up from two chemically identical moieties (called *A* and *B*), each one composed of two fused rings and connected through the C4*A*—C4*B* bond. The six-membered rings (C3*AA*/*AB*, C7*AA*/*AB*, C7*A*/*B*, C6*A*/*B*, C5*A*/*B*, C4*A*/*B*) adopt an envelope conformation with atoms C7*A*/*B* (located *para* to C4*A*/*B*) as the flap [puckering parameters are *Q* = 0.403 (2) Å, *θ* = 49.2 (3)°, *φ* = 108.2 (4)° and *Q* = 0.490 (2) Å, *θ* = 58.5 (2)°, *φ* = 114.9 (3)°, respectively]. The five-membered rings (O1*A*/*B*, C2*A*/*B*, O3*A*/*B*, C3*AA*/*AB*, C7*AA*/*AB*) adopt a twisted conformation [puckering parameters *Q*(2) = 0.3285 (17) Å, *φ*(2) = 115.6 (3)° and *Q*(2) = 0.3268 (18) Å, *φ*(2) = 101.4 (3)°, respectively]). In fragment *A*, the flap of the envelope is oriented away from the five-membered ring while in fragment *B*, both C7 and the five-membered ring are on the same side of the plane of the envelope, making them conformationally different. The dihedral angle between the least-square planes through the six-membered rings is 43.15 (9)° while the dihedral angles between the five and six-membered rings are 69.31 (10) and 76.95 (10)° in *A* and *B*, respectively, leaving the two five-membered rings on opposite sides of the C4*A*—C4*B* bond and almost in the same plane, normal to the bis­ector plane of both six-membered rings.

## Supra­molecular features   

**Figure 3:**
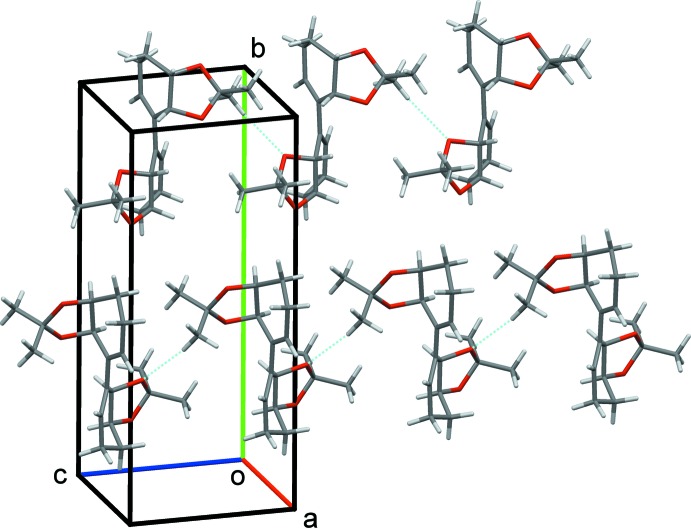
The crystal structure of the title compound, showing the C—H⋯O hydrogen-bonding inter­actions (dotted lines) along the [001] direction.

In the crystal, weak C22*A*—H22*F*⋯O3*B* ^i^ [symmetry code: (i) *x*, *y*, *z* − 1] inter­actions link the mol­ecules in chains running along [001], see Fig. 3 ▸ and Table 1 ▸. In the [100] and [010] directions, only weak dipolar inter­actions or van der Waals forces act between neighboring chains to stabilize the three-dimensional array of the crystal structure.

## Database survey   

A search of the Cambridge Structural Database (CSD Version 5.36 with one update; Groom *et al.*, 2016 ▸) using as a criterion the existence of mol­ecular structures composed of two similar fragments of fused five and six-membered rings gave no results. However, a search for similar systems considering only the six-membered ring resulted in four hits, *viz*. two different crystal structures for (5,5′-diphenyl-1,1′-bi(cyclo­hex-1-en-1-yl)-4,4′-di­yl)di­methanol in space groups *P*1 and *P* 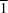, (*S*,*S*)-2,2′-bis­(di­phenyl­phosphino­yl)bi(cyclo­hex-1-ene) and (3*S*,6*R*)-3-isopropyl-2-[(3*R*,6*S*)-6-isopropyl-3-methyl-1-cyclo­hexen­yl]-6-methyl­cyclo­hexene; none of which is composed of fused rings. These results demonstrate the rarity of this sort of mol­ecule. While there are no reports about such systems, the structure of (3a*S*,4*S*,5*R*,7a*R*)-2,2,7-trimethyl-3a,4,5,7a-tetra­hydro-1,3-benzo­dioxole-4,5-diol was published recently (Macías *et al.*, 2015 ▸). In this case, the conformation of the fused rings keeps a level of similarity with the structural assembly of the title compound.

## Synthesis and crystallization   

A mixture of the vinylic iodide (mol­ecule **1** in Fig. 1 ▸.) (140 mg, 0.5 mmol), Pd(PPh_3_)_4_ (10% wt., 14.4 mg, 0.025 mmol), indium (28.7 mg, 0.25 mmol), and lithium chloride (31.8 mg, 0.75 mmol) in dry THF (2 mL) was stirred at reflux for 4 h under a nitro­gen atmosphere. The reaction mixture was quenched with NaHCO_3_ (sat. aq.). The aqueous layer was extracted with ethyl acetate (3 × 20 mL), and the combined organic phases were washed with brine, dried with Na_2_SO_4_, filtered and concentrated under reduced pressure. The residue was purified by silica gel column chromatography (hexa­nes/ethyl acetate 95:5) to give the desired homocoupled product (43.5 mg, 57%).

Crystals suitable for X-ray crystallographic analysis were obtained by dissolving the title compound in the minimum volume of ethyl acetate, adding hexa­nes until the solution became slightly turbid, and slowly evaporating the solvent at room temperature. ^1^H NMR (400 MHz, CDCl_3_) δ: 6.16 (*t*, *J* = 4.2 Hz, 2H), 4.72 (*d*, *J* = 5.6 Hz, 2H), 4.33–4.29 (*m*, 2H), 2.36–2.27 (*m*, 2H), 2.09–2.00 (*m*, 2H), 1.87–1.71 (*m*, 4H), 1.40 (*s*, 6H); 1.39 (*s*, 6H). All spectroscopic and analytical data were in full agreement with the literature (Boyd *et al.*, 2011 ▸).

## Refinement   

Crystal data, data collection and structure refinement details are summarized in Table 2 ▸. H atoms bonded to C were placed in calculated positions (C—H = 0.93–0.98 Å) and included as riding contributions with isotropic displacement parameters set to 1.2–1.5 times the *U*_eq_ of the parent atom.

## Supplementary Material

Crystal structure: contains datablock(s) I. DOI: 10.1107/S2056989016019927/rz5201sup1.cif

Structure factors: contains datablock(s) I. DOI: 10.1107/S2056989016019927/rz5201Isup2.hkl

CCDC reference: 1522804

Additional supporting information: crystallographic information; 3D view; checkCIF report

## Figures and Tables

**Table 1:** Hydrogen-bond geometry (Å, °)

*D*—H⋯*A*	*D*—H	H⋯*A*	*D*⋯*A*	*D*—H⋯*A*
C22*A*—H22*F*⋯O3*B* ^i^	0.96	2.56	3.510 (3)	171

Symmetry code: (i) 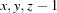.

**Table 2:** Experimental details

Crystal data	
Chemical formula	C_18_H_26_O_4_
*M*_r_	306.39
Crystal system, space group	Monoclinic, *P*2_1_
Temperature (K)	298
*a*, *b*, *c* (Å)	6.2927 (7), 17.9903 (19), 7.2991 (8)
β (°)	95.216 (4)
*V* (Å^3^)	822.89 (16)
*Z*	2
Radiation type	Cu *K*α
μ (mm^−1^)	0.69
Crystal size (mm)	0.40 × 0.35 × 0.30

Data collection	
Diffractometer	Bruker D8 Venture/Photon 100 CMOS
Absorption correction	Multi-scan (*SADABS*; Bruker, 2013 ▸)
*T*_min_, *T*_max_	0.687, 0.754
No. of measured, independent and observed [*I* > 2σ(*I*)] reflections	27011, 3232, 3135
*R*_int_	0.026
(sin θ/λ)_max_ (Å^−1^)	0.618

Refinement	
*R*[*F* ^2^ > 2σ(*F* ^2^)], *wR*(*F* ^2^), *S*	0.027, 0.071, 1.08
No. of reflections	3232
No. of parameters	204
No. of restraints	1
H-atom treatment	H-atom parameters constrained
Δρ_max_, Δρ_min_ (e Å^−3^)	0.13, −0.10
Absolute structure	Flack *x* determined using 1475 quotients [(*I* ^+^)−(*I* ^−^)]/[(*I* ^+^)+(*I* ^−^)] (Parsons *et al.*, 2013 ▸)
Absolute structure parameter	0.04 (4)

Computer programs: *APEX2* and *SAINT* (Bruker, 2013 ▸), *SHELXS2014* (Sheldrick, 2008 ▸, 2015 ▸), *SHELXL2014* (Sheldrick, 2015 ▸), *Mercury* (Macrae *et al.*, 2008 ▸) and *publCIF* (Westrip, 2010 ▸).

## supplementary crystallographic information

### Crystal data

**Table:**

C_18_H_26_O_4_	*F*(000) = 332
*M**_r_* = 306.39	*D*_x_ = 1.237 Mg m^−^^3^
Monoclinic, *P*2_1_	Cu *K*α radiation, λ = 1.54178 Å
*a* = 6.2927 (7) Å	Cell parameters from 9685 reflections
*b* = 17.9903 (19) Å	θ = 4.9–72.4°
*c* = 7.2991 (8) Å	µ = 0.69 mm^−^^1^
β = 95.216 (4)°	*T* = 298 K
*V* = 822.89 (16) Å^3^	Parallelepiped, yellow
*Z* = 2	0.40 × 0.35 × 0.30 mm

### Data collection

**Table:**

Bruker D8 Venture/Photon 100 CMOS diffractometer	3232 independent reflections
Radiation source: Cu Incoatec microsource	3135 reflections with *I* > 2σ(*I*)
Detector resolution: 10.4167 pixels mm^-1^	*R*_int_ = 0.026
φ and ω scans	θ_max_ = 72.4°, θ_min_ = 4.9°
Absorption correction: multi-scan (SADABS; Bruker, 2013)	*h* = −7→7
*T*_min_ = 0.687, *T*_max_ = 0.754	*k* = −21→22
27011 measured reflections	*l* = −9→9

### Refinement

**Table:**

Refinement on *F*^2^	Hydrogen site location: inferred from neighbouring sites
Least-squares matrix: full	H-atom parameters constrained
*R*[*F*^2^ > 2σ(*F*^2^)] = 0.027	*w* = 1/[σ^2^(*F*_o_^2^) + (0.0389*P*)^2^ + 0.0652*P*] where *P* = (*F*_o_^2^ + 2*F*_c_^2^)/3
*wR*(*F*^2^) = 0.071	(Δ/σ)_max_ < 0.001
*S* = 1.08	Δρ_max_ = 0.13 e Å^−^^3^
3232 reflections	Δρ_min_ = −0.10 e Å^−^^3^
204 parameters	Extinction correction: SHELXL, *F*_c_^*^ = *k**F*_c_[1+0.001*x**F*_c_^2^λ^3^/sin(2θ)]^-1/4^
1 restraint	Extinction coefficient: 0.0184 (15)
Primary atom site location: structure-invariant direct methods	Absolute structure: Flack *x* determined using 1475 quotients [(*I*^+^)-(*I*^-^)]/[(*I*^+^)+(*I*^-^)] (Parsons *et al.*, 2013)
Secondary atom site location: difference Fourier map	Absolute structure parameter: 0.04 (4)

### Special details

**Table:**

Geometry. All esds (except the esd in the dihedral angle between two l.s. planes) are estimated using the full covariance matrix. The cell esds are taken into account individually in the estimation of esds in distances, angles and torsion angles; correlations between esds in cell parameters are only used when they are defined by crystal symmetry. An approximate (isotropic) treatment of cell esds is used for estimating esds involving l.s. planes.

### Fractional atomic coordinates and isotropic or equivalent isotropic displacement parameters (Å^2^)

**Table:**

	*x*	*y*	*z*	*U*_iso_*/*U*_eq_	
C7A	0.6166 (3)	1.13949 (11)	0.8000 (3)	0.0486 (4)	
H7AA	0.5916	1.1910	0.7652	0.058*	
H7AB	0.6639	1.1382	0.9302	0.058*	
C6B	0.5034 (4)	0.77162 (12)	0.6693 (3)	0.0621 (6)	
H6BA	0.5554	0.7523	0.5577	0.074*	
H6BB	0.3603	0.7526	0.6774	0.074*	
C2A	0.8127 (3)	1.05458 (10)	0.4052 (2)	0.0451 (4)	
C7B	0.6472 (4)	0.74545 (11)	0.8343 (3)	0.0553 (5)	
H7BA	0.5835	0.7580	0.9464	0.066*	
H7BB	0.6625	0.6919	0.8297	0.066*	
C22A	0.6299 (3)	1.01769 (13)	0.2920 (3)	0.0580 (5)	
H22D	0.5609	1.0532	0.2084	0.087*	
H22E	0.5291	0.9991	0.3719	0.087*	
H22F	0.6832	0.9773	0.2235	0.087*	
C2B	1.0094 (3)	0.83055 (10)	1.1168 (3)	0.0480 (4)	
C21A	0.9882 (4)	1.07986 (15)	0.2908 (3)	0.0674 (6)	
H21D	1.0446	1.0377	0.2306	0.101*	
H21E	1.1000	1.1030	0.3691	0.101*	
H21F	0.9314	1.1149	0.2000	0.101*	
C21B	1.2355 (4)	0.85972 (14)	1.1342 (4)	0.0659 (6)	
H21A	1.2803	0.8687	1.0139	0.099*	
H21B	1.3284	0.8237	1.1970	0.099*	
H21C	1.2417	0.9053	1.2029	0.099*	
C22B	0.9298 (5)	0.81210 (16)	1.2994 (3)	0.0716 (7)	
H22A	0.7901	0.7905	1.2800	0.107*	
H22B	0.9231	0.8567	1.3710	0.107*	
H22C	1.0254	0.7774	1.3637	0.107*	
C6A	0.4092 (3)	1.09664 (11)	0.7669 (3)	0.0548 (5)	
H6AA	0.3159	1.1102	0.8602	0.066*	
H6AB	0.3389	1.1105	0.6481	0.066*	
C5B	0.4969 (3)	0.85505 (11)	0.6607 (3)	0.0491 (4)	
H5B	0.3814	0.8774	0.5935	0.059*	
C5A	0.4430 (3)	1.01436 (10)	0.7714 (3)	0.0452 (4)	
H5A	0.3277	0.9845	0.7940	0.054*	
C4B	0.6466 (3)	0.89895 (9)	0.7434 (2)	0.0363 (3)	
C4A	0.6263 (2)	0.98093 (9)	0.7455 (2)	0.0354 (4)	
C3AB	0.8491 (3)	0.86610 (9)	0.8348 (2)	0.0370 (4)	
H3AB	0.9718	0.8867	0.7785	0.044*	
O3B	0.8671 (2)	0.88358 (7)	1.02685 (17)	0.0462 (3)	
C3AA	0.8247 (2)	1.02504 (9)	0.7153 (2)	0.0355 (3)	
H3AA	0.9338	1.0159	0.8173	0.043*	
O3A	0.90773 (18)	1.00550 (6)	0.54514 (17)	0.0426 (3)	
C7AB	0.8628 (3)	0.78135 (10)	0.8361 (2)	0.0452 (4)	
H7B	0.9330	0.7645	0.7290	0.054*	
O1B	0.9974 (3)	0.76615 (7)	1.0002 (2)	0.0601 (4)	
C7AA	0.7894 (3)	1.10842 (9)	0.6933 (3)	0.0423 (4)	
H7A	0.9235	1.1349	0.7263	0.051*	
O1A	0.7303 (3)	1.11563 (8)	0.50051 (18)	0.0580 (4)	

### Atomic displacement parameters (Å^2^)

**Table:**

	*U*^11^	*U*^22^	*U*^33^	*U*^12^	*U*^13^	*U*^23^
C7A	0.0564 (11)	0.0367 (9)	0.0508 (10)	0.0081 (8)	−0.0048 (8)	−0.0077 (8)
C6B	0.0703 (14)	0.0432 (11)	0.0720 (14)	−0.0180 (10)	0.0032 (11)	−0.0097 (10)
C2A	0.0536 (10)	0.0381 (9)	0.0439 (9)	0.0048 (8)	0.0056 (8)	0.0063 (7)
C7B	0.0742 (13)	0.0309 (9)	0.0634 (12)	−0.0063 (8)	0.0210 (10)	0.0003 (8)
C22A	0.0607 (11)	0.0618 (13)	0.0506 (11)	0.0013 (10)	−0.0001 (9)	−0.0027 (9)
C2B	0.0613 (11)	0.0383 (9)	0.0438 (9)	0.0127 (8)	0.0026 (8)	0.0052 (7)
C21A	0.0667 (14)	0.0740 (15)	0.0628 (13)	−0.0058 (11)	0.0129 (10)	0.0236 (11)
C21B	0.0605 (13)	0.0570 (13)	0.0785 (15)	0.0122 (10)	−0.0026 (11)	0.0089 (11)
C22B	0.0899 (17)	0.0771 (17)	0.0488 (12)	0.0198 (14)	0.0117 (11)	0.0137 (11)
C6A	0.0462 (10)	0.0513 (12)	0.0652 (12)	0.0139 (8)	−0.0034 (8)	−0.0090 (9)
C5B	0.0524 (10)	0.0445 (10)	0.0495 (10)	−0.0073 (8)	−0.0002 (8)	0.0003 (8)
C5A	0.0372 (8)	0.0459 (10)	0.0521 (10)	−0.0014 (7)	0.0022 (7)	−0.0023 (8)
C4B	0.0419 (8)	0.0333 (8)	0.0342 (8)	−0.0025 (6)	0.0060 (6)	0.0024 (6)
C4A	0.0371 (8)	0.0348 (8)	0.0334 (8)	−0.0008 (6)	−0.0019 (6)	0.0006 (6)
C3AB	0.0435 (8)	0.0295 (8)	0.0386 (8)	0.0007 (6)	0.0074 (6)	0.0005 (6)
O3B	0.0590 (7)	0.0379 (6)	0.0405 (6)	0.0146 (6)	−0.0024 (5)	−0.0036 (5)
C3AA	0.0347 (7)	0.0317 (8)	0.0391 (8)	0.0016 (6)	−0.0025 (6)	0.0019 (6)
O3A	0.0453 (6)	0.0363 (6)	0.0472 (6)	0.0065 (5)	0.0097 (5)	0.0063 (5)
C7AB	0.0606 (11)	0.0331 (9)	0.0433 (9)	0.0062 (8)	0.0133 (8)	−0.0010 (7)
O1B	0.0851 (9)	0.0355 (7)	0.0581 (8)	0.0181 (7)	−0.0034 (7)	0.0022 (6)
C7AA	0.0477 (9)	0.0297 (8)	0.0481 (9)	−0.0021 (7)	−0.0038 (7)	0.0010 (7)
O1A	0.0899 (10)	0.0370 (7)	0.0464 (7)	0.0179 (7)	0.0028 (7)	0.0078 (5)

### Geometric parameters (Å, º)

**Table:**

C7A—C7AA	1.502 (3)	C21B—H21B	0.9600
C7A—C6A	1.516 (3)	C21B—H21C	0.9600
C7A—H7AA	0.9700	C22B—H22A	0.9600
C7A—H7AB	0.9700	C22B—H22B	0.9600
C6B—C5B	1.503 (3)	C22B—H22C	0.9600
C6B—C7B	1.514 (3)	C6A—C5A	1.495 (3)
C6B—H6BA	0.9700	C6A—H6AA	0.9700
C6B—H6BB	0.9700	C6A—H6AB	0.9700
C2A—O1A	1.423 (2)	C5B—C4B	1.331 (3)
C2A—O3A	1.439 (2)	C5B—H5B	0.9300
C2A—C22A	1.508 (3)	C5A—C4A	1.329 (2)
C2A—C21A	1.514 (3)	C5A—H5A	0.9300
C7B—C7AB	1.501 (3)	C4B—C4A	1.481 (2)
C7B—H7BA	0.9700	C4B—C3AB	1.505 (2)
C7B—H7BB	0.9700	C4A—C3AA	1.512 (2)
C22A—H22D	0.9600	C3AB—O3B	1.431 (2)
C22A—H22E	0.9600	C3AB—C7AB	1.527 (2)
C22A—H22F	0.9600	C3AB—H3AB	0.9800
C2B—O3B	1.427 (2)	C3AA—O3A	1.434 (2)
C2B—O1B	1.435 (2)	C3AA—C7AA	1.523 (2)
C2B—C22B	1.502 (3)	C3AA—H3AA	0.9800
C2B—C21B	1.511 (3)	C7AB—O1B	1.430 (2)
C21A—H21D	0.9600	C7AB—H7B	0.9800
C21A—H21E	0.9600	C7AA—O1A	1.428 (2)
C21A—H21F	0.9600	C7AA—H7A	0.9800
C21B—H21A	0.9600		
			
C7AA—C7A—C6A	112.42 (16)	H22A—C22B—H22B	109.5
C7AA—C7A—H7AA	109.1	C2B—C22B—H22C	109.5
C6A—C7A—H7AA	109.1	H22A—C22B—H22C	109.5
C7AA—C7A—H7AB	109.1	H22B—C22B—H22C	109.5
C6A—C7A—H7AB	109.1	C5A—C6A—C7A	112.39 (15)
H7AA—C7A—H7AB	107.9	C5A—C6A—H6AA	109.1
C5B—C6B—C7B	110.85 (17)	C7A—C6A—H6AA	109.1
C5B—C6B—H6BA	109.5	C5A—C6A—H6AB	109.1
C7B—C6B—H6BA	109.5	C7A—C6A—H6AB	109.1
C5B—C6B—H6BB	109.5	H6AA—C6A—H6AB	107.9
C7B—C6B—H6BB	109.5	C4B—C5B—C6B	123.93 (18)
H6BA—C6B—H6BB	108.1	C4B—C5B—H5B	118.0
O1A—C2A—O3A	105.85 (14)	C6B—C5B—H5B	118.0
O1A—C2A—C22A	108.30 (16)	C4A—C5A—C6A	124.64 (17)
O3A—C2A—C22A	111.49 (15)	C4A—C5A—H5A	117.7
O1A—C2A—C21A	110.76 (17)	C6A—C5A—H5A	117.7
O3A—C2A—C21A	107.39 (16)	C5B—C4B—C4A	122.49 (16)
C22A—C2A—C21A	112.85 (18)	C5B—C4B—C3AB	120.26 (16)
C7AB—C7B—C6B	110.32 (16)	C4A—C4B—C3AB	117.22 (14)
C7AB—C7B—H7BA	109.6	C5A—C4A—C4B	121.95 (15)
C6B—C7B—H7BA	109.6	C5A—C4A—C3AA	121.45 (15)
C7AB—C7B—H7BB	109.6	C4B—C4A—C3AA	116.60 (14)
C6B—C7B—H7BB	109.6	O3B—C3AB—C4B	109.67 (13)
H7BA—C7B—H7BB	108.1	O3B—C3AB—C7AB	102.38 (13)
C2A—C22A—H22D	109.5	C4B—C3AB—C7AB	116.09 (15)
C2A—C22A—H22E	109.5	O3B—C3AB—H3AB	109.5
H22D—C22A—H22E	109.5	C4B—C3AB—H3AB	109.5
C2A—C22A—H22F	109.5	C7AB—C3AB—H3AB	109.5
H22D—C22A—H22F	109.5	C2B—O3B—C3AB	107.11 (13)
H22E—C22A—H22F	109.5	O3A—C3AA—C4A	111.61 (13)
O3B—C2B—O1B	105.65 (14)	O3A—C3AA—C7AA	102.20 (13)
O3B—C2B—C22B	108.06 (17)	C4A—C3AA—C7AA	114.69 (14)
O1B—C2B—C22B	110.10 (19)	O3A—C3AA—H3AA	109.4
O3B—C2B—C21B	110.69 (17)	C4A—C3AA—H3AA	109.4
O1B—C2B—C21B	109.18 (18)	C7AA—C3AA—H3AA	109.4
C22B—C2B—C21B	112.9 (2)	C3AA—O3A—C2A	107.67 (12)
C2A—C21A—H21D	109.5	O1B—C7AB—C7B	113.01 (16)
C2A—C21A—H21E	109.5	O1B—C7AB—C3AB	102.98 (15)
H21D—C21A—H21E	109.5	C7B—C7AB—C3AB	112.28 (15)
C2A—C21A—H21F	109.5	O1B—C7AB—H7B	109.5
H21D—C21A—H21F	109.5	C7B—C7AB—H7B	109.5
H21E—C21A—H21F	109.5	C3AB—C7AB—H7B	109.5
C2B—C21B—H21A	109.5	C7AB—O1B—C2B	109.73 (13)
C2B—C21B—H21B	109.5	O1A—C7AA—C7A	109.95 (15)
H21A—C21B—H21B	109.5	O1A—C7AA—C3AA	102.46 (14)
C2B—C21B—H21C	109.5	C7A—C7AA—C3AA	114.67 (15)
H21A—C21B—H21C	109.5	O1A—C7AA—H7A	109.8
H21B—C21B—H21C	109.5	C7A—C7AA—H7A	109.8
C2B—C22B—H22A	109.5	C3AA—C7AA—H7A	109.8
C2B—C22B—H22B	109.5	C2A—O1A—C7AA	109.73 (13)
			
C5B—C6B—C7B—C7AB	−52.6 (2)	C7AA—C3AA—O3A—C2A	−31.87 (16)
C7AA—C7A—C6A—C5A	44.4 (2)	O1A—C2A—O3A—C3AA	17.72 (18)
C7B—C6B—C5B—C4B	21.4 (3)	C22A—C2A—O3A—C3AA	−99.84 (17)
C7A—C6A—C5A—C4A	−21.2 (3)	C21A—C2A—O3A—C3AA	136.07 (17)
C6B—C5B—C4B—C4A	−174.59 (18)	C6B—C7B—C7AB—O1B	172.70 (16)
C6B—C5B—C4B—C3AB	7.3 (3)	C6B—C7B—C7AB—C3AB	56.7 (2)
C6A—C5A—C4A—C4B	−177.19 (17)	O3B—C3AB—C7AB—O1B	−31.08 (16)
C6A—C5A—C4A—C3AA	2.4 (3)	C4B—C3AB—C7AB—O1B	−150.49 (15)
C5B—C4B—C4A—C5A	41.2 (3)	O3B—C3AB—C7AB—C7B	90.79 (17)
C3AB—C4B—C4A—C5A	−140.59 (17)	C4B—C3AB—C7AB—C7B	−28.6 (2)
C5B—C4B—C4A—C3AA	−138.43 (17)	C7B—C7AB—O1B—C2B	−104.65 (19)
C3AB—C4B—C4A—C3AA	39.8 (2)	C3AB—C7AB—O1B—C2B	16.7 (2)
C5B—C4B—C3AB—O3B	−119.03 (18)	O3B—C2B—O1B—C7AB	4.1 (2)
C4A—C4B—C3AB—O3B	62.73 (18)	C22B—C2B—O1B—C7AB	120.57 (19)
C5B—C4B—C3AB—C7AB	−3.7 (2)	C21B—C2B—O1B—C7AB	−114.94 (18)
C4A—C4B—C3AB—C7AB	178.09 (14)	C6A—C7A—C7AA—O1A	63.7 (2)
O1B—C2B—O3B—C3AB	−25.2 (2)	C6A—C7A—C7AA—C3AA	−51.0 (2)
C22B—C2B—O3B—C3AB	−143.02 (19)	O3A—C3AA—C7AA—O1A	33.75 (16)
C21B—C2B—O3B—C3AB	92.87 (19)	C4A—C3AA—C7AA—O1A	−87.19 (17)
C4B—C3AB—O3B—C2B	158.69 (15)	O3A—C3AA—C7AA—C7A	152.83 (14)
C7AB—C3AB—O3B—C2B	34.88 (17)	C4A—C3AA—C7AA—C7A	31.9 (2)
C5A—C4A—C3AA—O3A	−122.95 (16)	O3A—C2A—O1A—C7AA	5.2 (2)
C4B—C4A—C3AA—O3A	56.69 (18)	C22A—C2A—O1A—C7AA	124.91 (17)
C5A—C4A—C3AA—C7AA	−7.3 (2)	C21A—C2A—O1A—C7AA	−110.85 (19)
C4B—C4A—C3AA—C7AA	172.30 (14)	C7A—C7AA—O1A—C2A	−146.62 (16)
C4A—C3AA—O3A—C2A	91.17 (15)	C3AA—C7AA—O1A—C2A	−24.27 (19)

### Hydrogen-bond geometry (Å, º)

**Table:**

*D*—H···*A*	*D*—H	H···*A*	*D*···*A*	*D*—H···*A*
C22*A*—H22*F*···O3*B*^i^	0.96	2.56	3.510 (3)	171

Symmetry code: (i) *x*, *y*, *z*−1.
